# Supplementary material for: Bound states at partial dislocation defects in multipole higher-order topological insulators
Source: Nat Commun. 2022 Apr 19;13:2035. doi: 10.1038/s41467-022-29785-5 (PMC9018851; doi:10.1038/s41467-022-29785-5)
Supplement: Supplementary file 1 — Supplementary Information [file 41467_2022_29785_MOESM1_ESM.pdf]

Supplementary Materials: Bound states at partial dislocation defects in multipole higher-order topological insulators

Sasha S. Yamada[1], Tianhe Li[2], Mao Lin[2], Christopher W. Peterson[1], Taylor L. Hughes[2], and Gaurav Bahl[3]

[1]Department of Electrical and Computer Engineering,

[2]Department of Physics and Institute for Condensed Matter Theory,
[3]Department of Mechanical Science and Engineering,

University of Illinois at Urbana-Champaign, Urbana, IL, USA

S1 Procedure for determining the presence of trapped modes at partial dislocations

Here we provide a detailed agnostic procedure using the theoretical framework of embedded topological insulators [1] for determining whether a partial dislocation will trap a topological bound state and how to develop an index theorem.

1. Start with a D-dimensional lattice model which has some degrees of freedom in the unit cell that are spatially separated.

2. Fill the system up to an energy gap and then determine the filled-band ground state.

3. Next make a spatial entanglement cut that, instead of cutting between whole unit cells as is conventionally done, cuts through unit cells. For example, if one had a Su-Schrieffer-Heeger dimerized chain one could make an entanglement cut that splits a unit cell in half, or in the Benalcazar-Bernevig-Hughes model [2] it would separate one of the internal Su-Schrieffer-Heeger chains from the other.

4. If the resulting single-particle entanglement spectrum is gapped (i.e., the many-body entanglement spectrum has a unique ground state), one can take the ground state of the many-body entanglement Hamiltonian and calculate any relevant (D-1)-dimensional topological invariants for the (D-1)-dimensional space transverse to the cut.

5. If any of these invariants are non-trivial then one can conclude that the (D-1)-dimensional gapped system near the entanglement cut is a (D-1)-topological insulator. If the entanglement cut was made in such a way that cutting through the unit cell would lead to a stacking fault, then one concludes that such stacking faults would be (D-1)-topological insulators.

6. Now one can apply the full machinery of the (D-1)-dimensional index theorems to determine the bulk boundary correspondence. That is, when one cuts the stacking fault to generate a partial dislocation, the (D-1)-dimensional bulk boundary correspondence will indicate if there is a localized fractional charge and/or bound state protected by: (i) the bulk gap (so the bound state does not leak off the entanglement-cut), (ii) the gap along the stacking fault (so the bound state does not delocalize along the fault), and (iii) any symmetries necessitated by the relevant topological invariants (e.g., mirror symmetry, time-reversal, chiral symmetry).

This procedure is similar to the nested Wilson loop/nested entanglement techniques used to identify boundary obstructed higher order topological insulators, but the difference here is that the entanglement cut is happening inside the unit cell instead of between unit cells. The part that connects to partial dislocations, and the embedding of the degrees of freedom in the unit cell, is in step 5 where the way we cut into the unit cell is identified with a particular

stacking fault. We emphasize that this procedure is generic as long as one has a crystal structure where various types of stacking faults can be identified a priori from the crystal structure, so that one knows what types of entanglement cuts to make.

If we apply this procedure to the Benalcazar-Bernevig-Hughes quadrupole model [2] we would find embedded topology protected by, for example, mirror symmetry. The embedded topology matches that of a mirror-symmetric Su-Schrieffer-Heeger model. A stacking fault represented by an odd number of Su-Schrieffer-Heeger chains would then have a non-trivial $\mathbb{Z}_2$ topology that will generate a fractional $1/2$ charge on a partial dislocation and the possibility of a localized topological bound state that can appear in the spectral gap. If we now apply it to the octupole model we find that the octupole model has embedded quadrupolar topology, i.e., has an embedded topology that matches the ordinary topology of the 2D quadrupole model.

References

[1] Tuegel, T. I., Chua, V. & Hughes, T. L. Embedded topological insulators. *Physical Review B* **100**, 115126 (2019).

[2] Benalcazar, W. A., Bernevig, B. A. & Hughes, T. L. Quantized electric multipole insulators. *Science* **357**, 61–66 (2017).

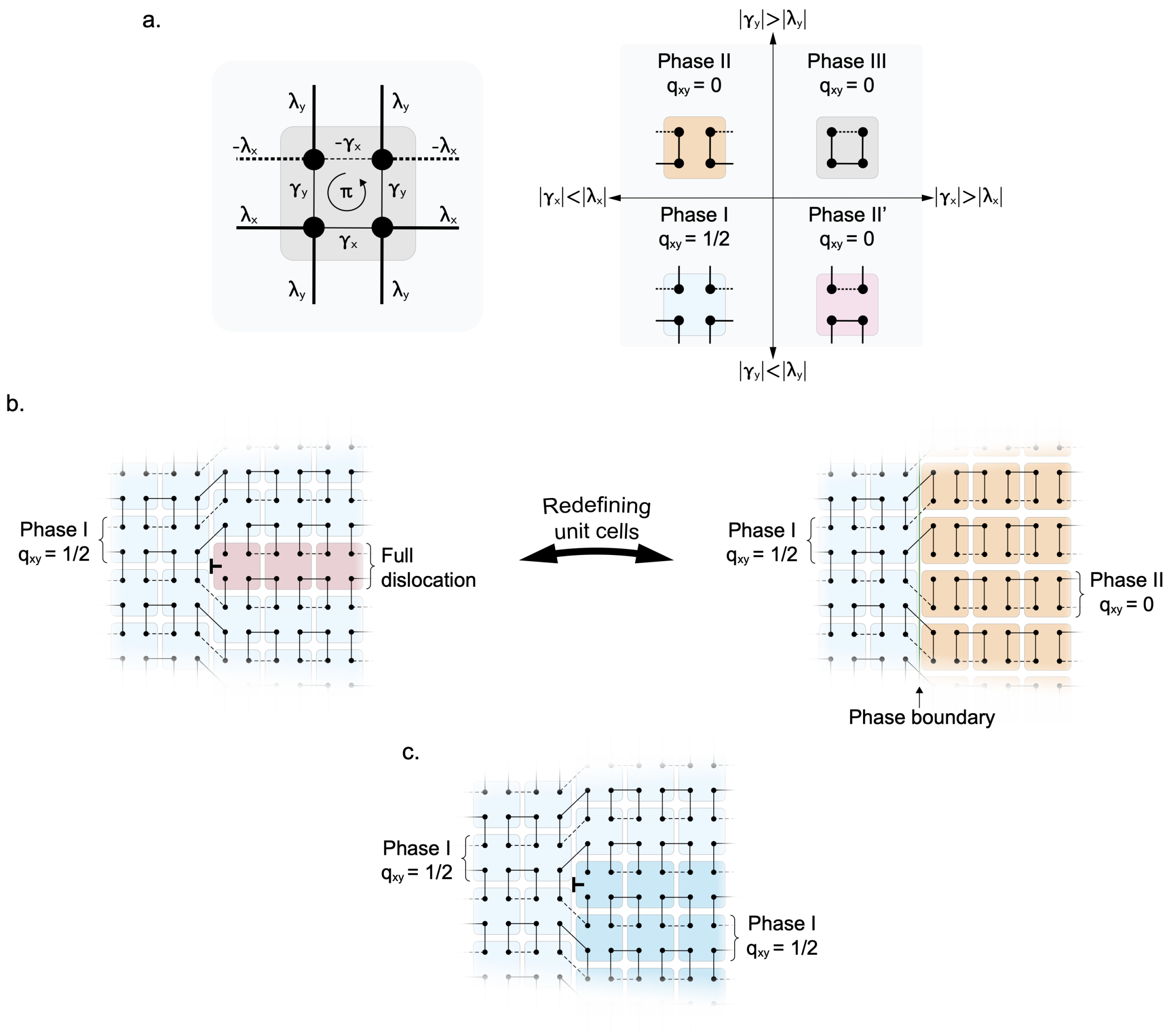

Figure S1: Showing that full dislocations are featureless at defect core. **(a)** Possible connections in the minimal model of a single quadrupolar topological crystalline insulator (TCI) unit cell and corresponding phase diagram. Intra-cell couplings are labeled as $\gamma_{x,y}$ and inter-cell couplings are labeled as $\lambda_{x,y}$. The dashed lines denote negative coupling. In the phase diagram, the dimerized limit is illustrated, so only the dominant connections are shown. **(b)** 2D quadrupolar TCI (blue unit cells) with inserted full dislocation (red unit cells). Redefining the unit cells of the lattice indeed yields a phase boundary, but does not trap any 0D bound states as there is no higher-order boundary between the two phases. Again, the dimerized limit is illustrated here. **(c)** Attempting to redefine the unit cells in a single quadrant yields a trivial result, as both regions are in the same topological phase.

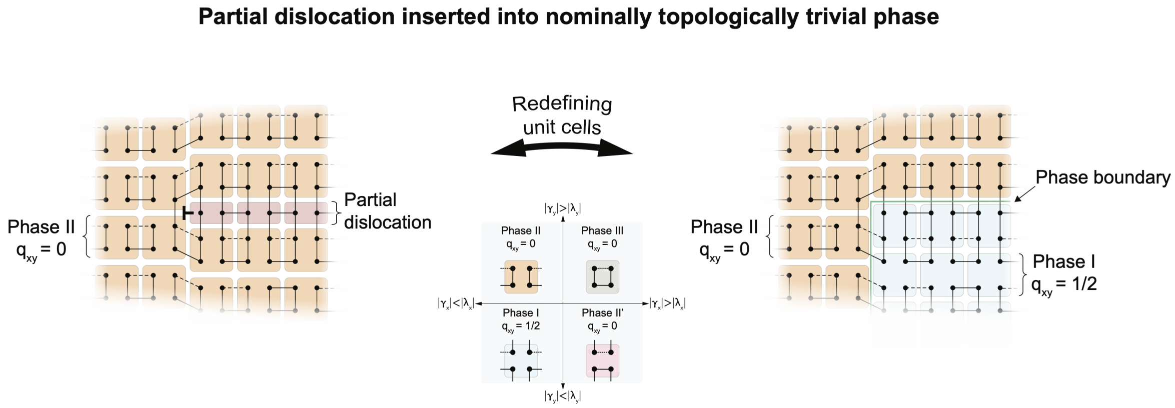

Figure S2: Illustration of partial dislocation defect in a nominally topologically trivial lattice. A partial dislocation oriented along $\hat{\mathbf{x}}$-direction of a nominally trivial quadrupolar TCI (converse of the case studied in main text) also forms a phase boundary and thus traps a bound state at the higher-order point (i.e., corner) of that boundary. It should be noted that a partial dislocation oriented along $\hat{\mathbf{y}}$-direction will yield a phase boundary between phase II and phase III unit cells. In this case, the quantized quadrupole moment per unit cell is zero for both phases, hence no bound states will be trapped at the partial dislocation defect core.

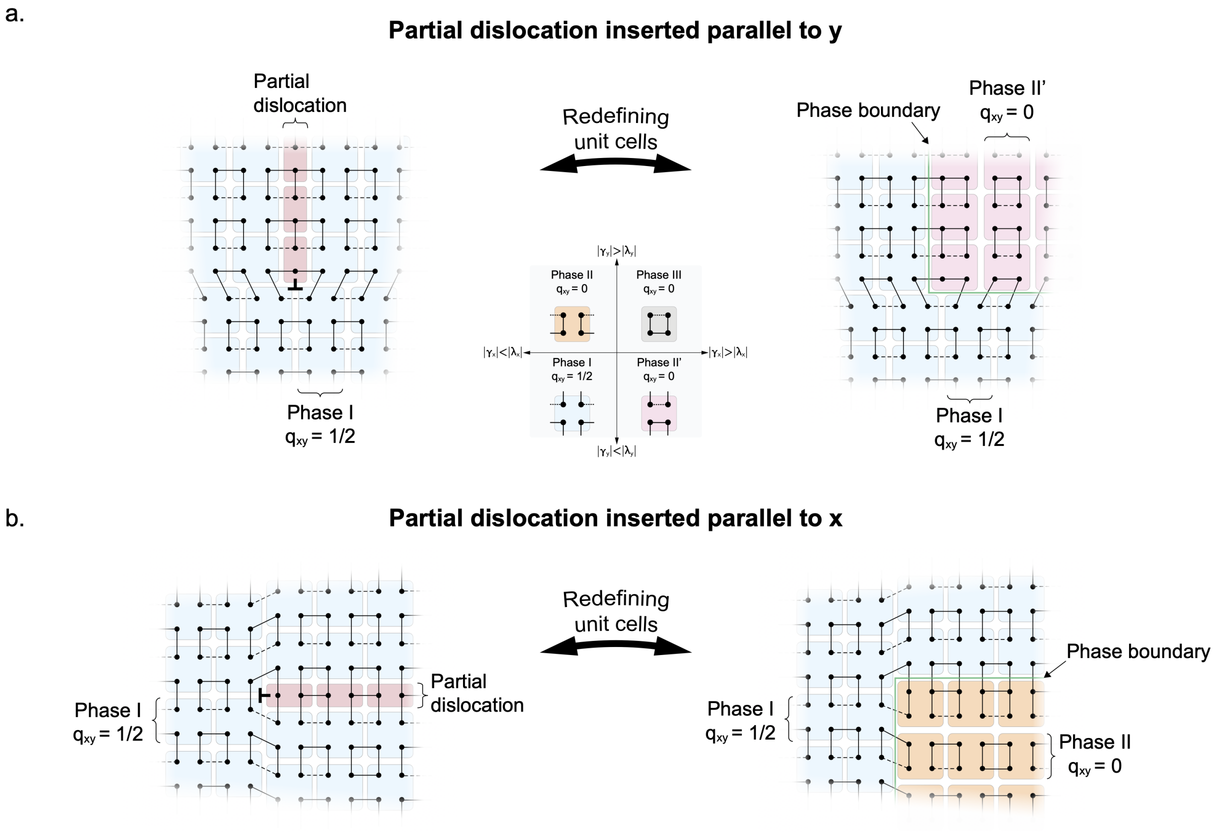

Figure S3: Visualization of criteria for partial dislocation to probe the higher-order topology of a quadrupolar TCI. (a) Partial dislocation oriented along $\hat{\mathbf{y}}$-direction, which forms a phase boundary between topologically trivial (phase II’) and non-trivial (phase I) regions. Since the quantized quadrupole moment per unit cell is different in these two regions, a bound state will be trapped at the higher-order point of their phase boundary. **(b)** Partial dislocation oriented along $\hat{\mathbf{x}}$-direction of quadrupolar TCI (case studied in main text). For the same reasons as (a), this defect also traps a bound state. Since the quadrupole TCI in phase I traps bound states at partial dislocations oriented along both the $\hat{\mathbf{x}}$- and $\hat{\mathbf{y}}$- directions, it can be concluded that the insulator is a HOTI.

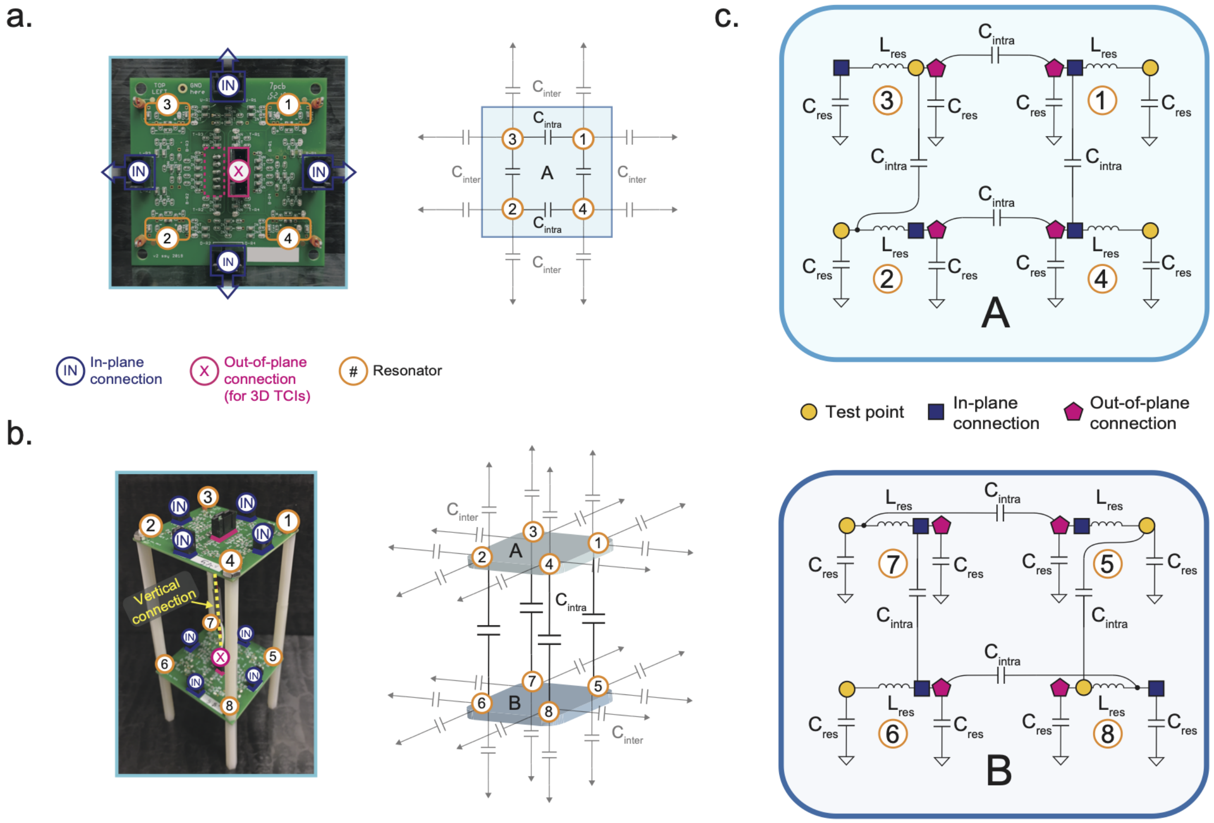

Figure S4: Details of circuit implementation. (a) Photo of quadrupolar TCI unit cell and illustration of inter-cell connectivity **(b)** same as (a) but for octupolar TCI **(c)** Schematic of circuit used to realize a single octupolar TCI unit cell with 8 sites. The sub-units A and B each correspond to one modular circuit board. Only the A sub-units were used to realize the 2D quadrupolar TCI. The nodes used as measurement test points are labeled with yellow circles. In-plane connections to neighboring circuit boards were made at the nodes labeled by the green squares. Out-of-plane connections were made at the nodes closest to the center of each circuit board and are labeled with purple pentagons. The component values used were: $C_{res} = 200$ pF, $L_{res} = 1$ μF, $C_{intra} = 10$ pF, and $C_{inter} = 100$ pF

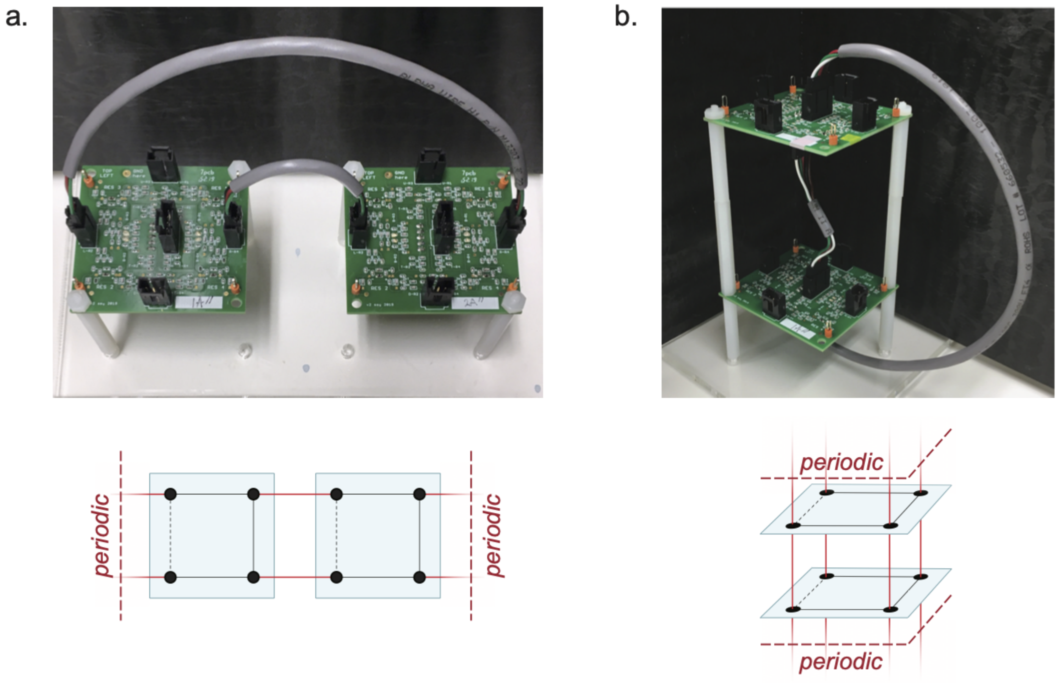

Figure S5: Implementing periodic boundary conditions. **(a)** Example of in-plane and **(b)** out-of-plane periodic boundary conditions using two four-site unit cells. Periodicity is enforced by physically wiring the two unit cells together.

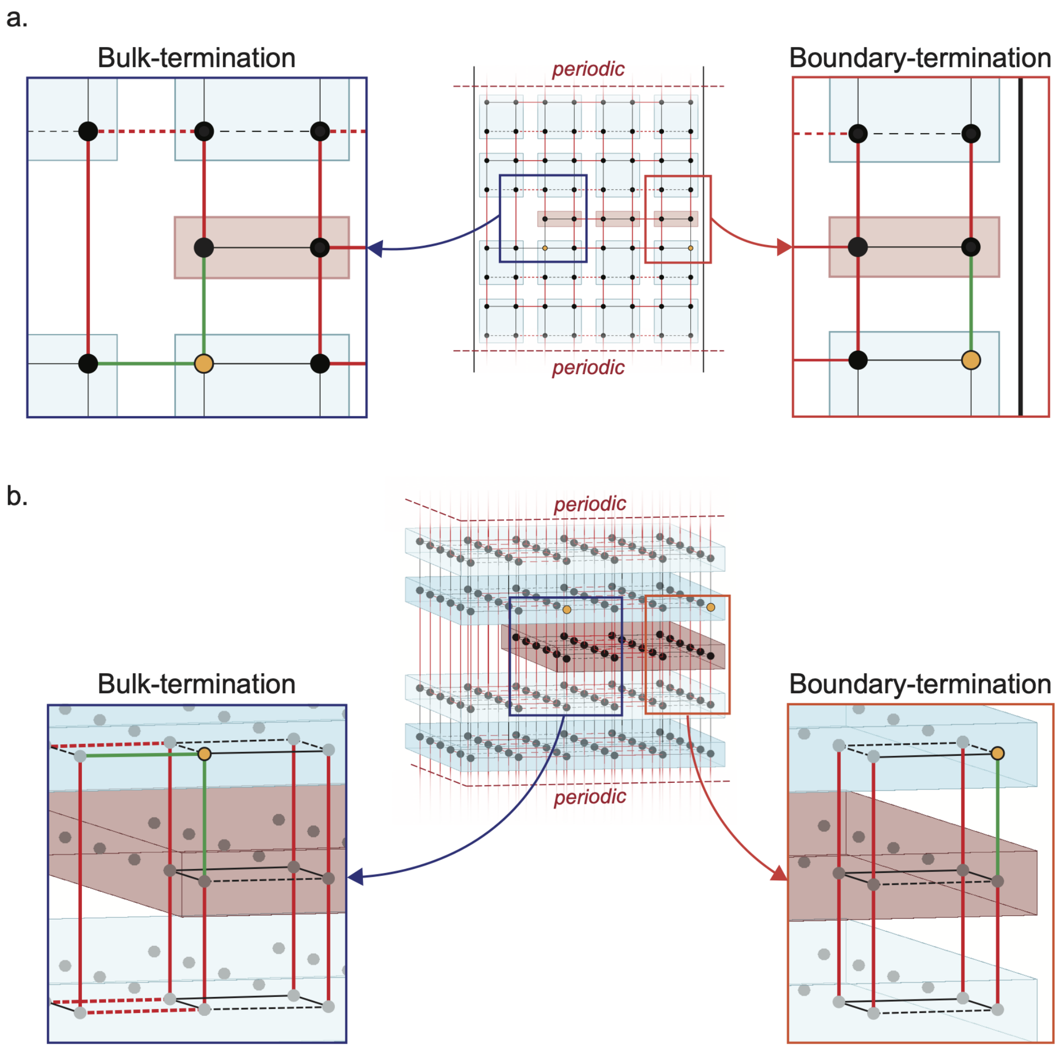

Figure S6: Local deformation of coupling to spatially isolate bound states to single sites. **(a)** Representative examples of how the coupling pattern was locally deformed to spatially isolate the bound states associated with bulk- and boundary-terminated partial dislocation defects to a single site in 2D quadrupolar topological crystalline insulators (TCIs). Additional explanations are provided in Figs. S13–S17. The red (black) lines denote strong (weak) coupling, and the dashed lines denote negative coupling. The green lines correspond to where the coupling strength was changed from strong to weak coupling in order to isolate a bound state on the site highlighted in yellow. **(b)** same as (a) but for partial dislocation plane defects in 3D octupolar TCIs.

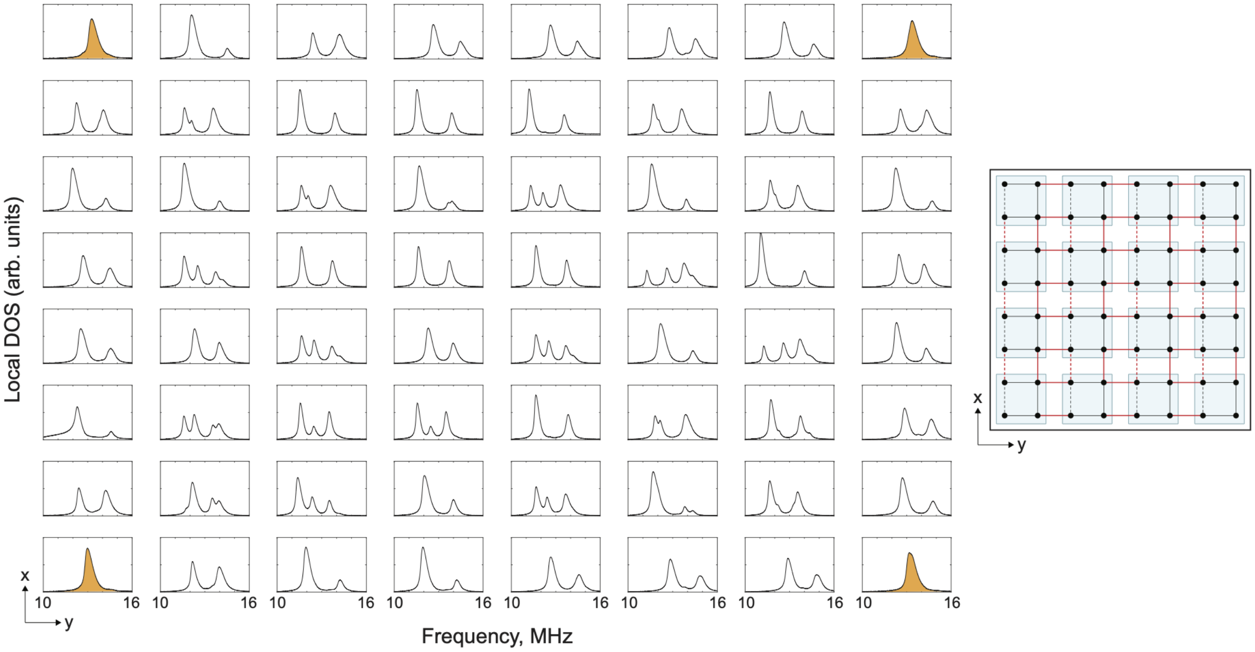

Figure S7: Measured local DOS at each site for a 4×4 unit-cell quadrupolar TCI. These measurements correspond to main manuscript Fig. 2c. The organization of the grid follows the topology on the right, where the red (black) lines denote strong (weak) coupling, and the dashed lines denote negative coupling. In the DOS measurements, sites that are anticipated to host a corner mode or bound state are highlighted in yellow.

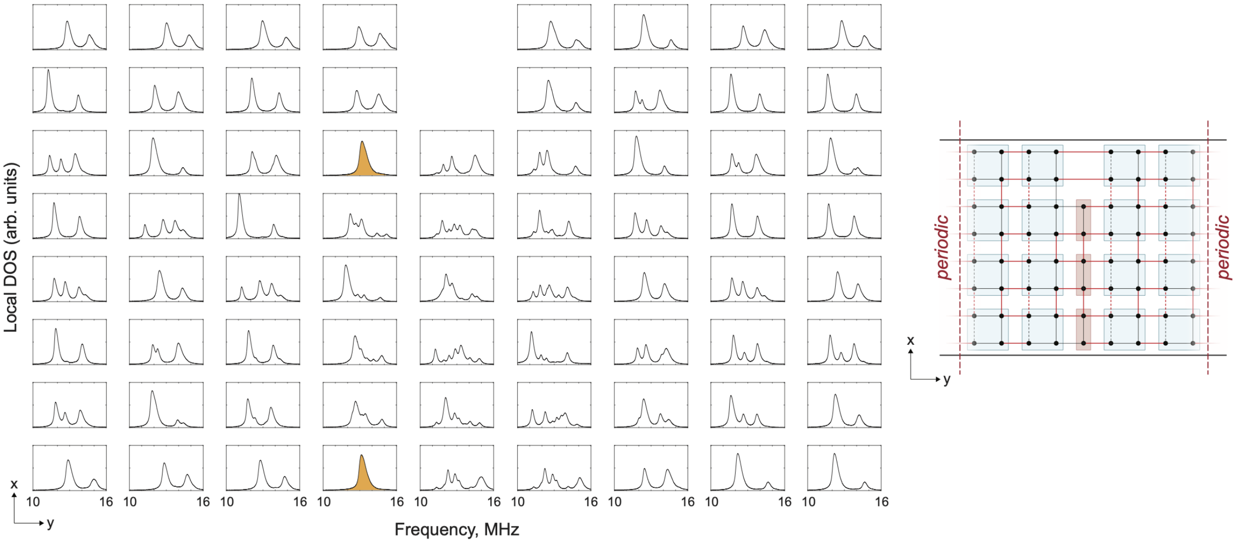

Figure S8: Measured local DOS at each site for a 4×4 unit-cell quadrupolar TCI with a bulk-terminated partial dislocation. These measurements correspond to main manuscript Fig. 3a. The organization of the grid follows the topology on the right, the red (black) lines denote strong (weak) coupling, and the dashed lines denote negative coupling. In the DOS measurements, sites that are anticipated to host a corner mode or bound state are highlighted in yellow. An agnostic approach to identifying the sites of interest is provided in Figs. S13–S17.

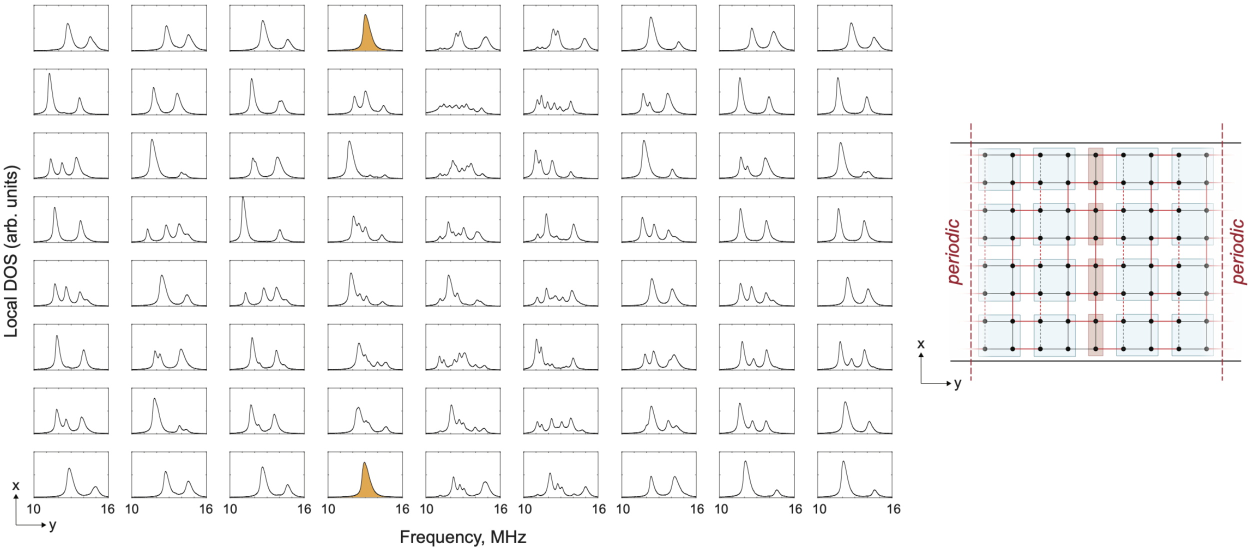

Figure S9: Measured local DOS at each site for a 4×4 unit-cell quadrupolar TCI with a boundary-terminated partial dislocation. These measurements correspond to main manuscript Fig. 3b. The organization of the grid follows the topology on the right, the red (black) lines denote strong (weak) coupling, and the dashed lines denote negative coupling. In the DOS measurements, sites that are anticipated to host a corner mode or bound state are highlighted in yellow. An agnostic approach to identifying the sites of interest is provided in Figs. S13–S17.

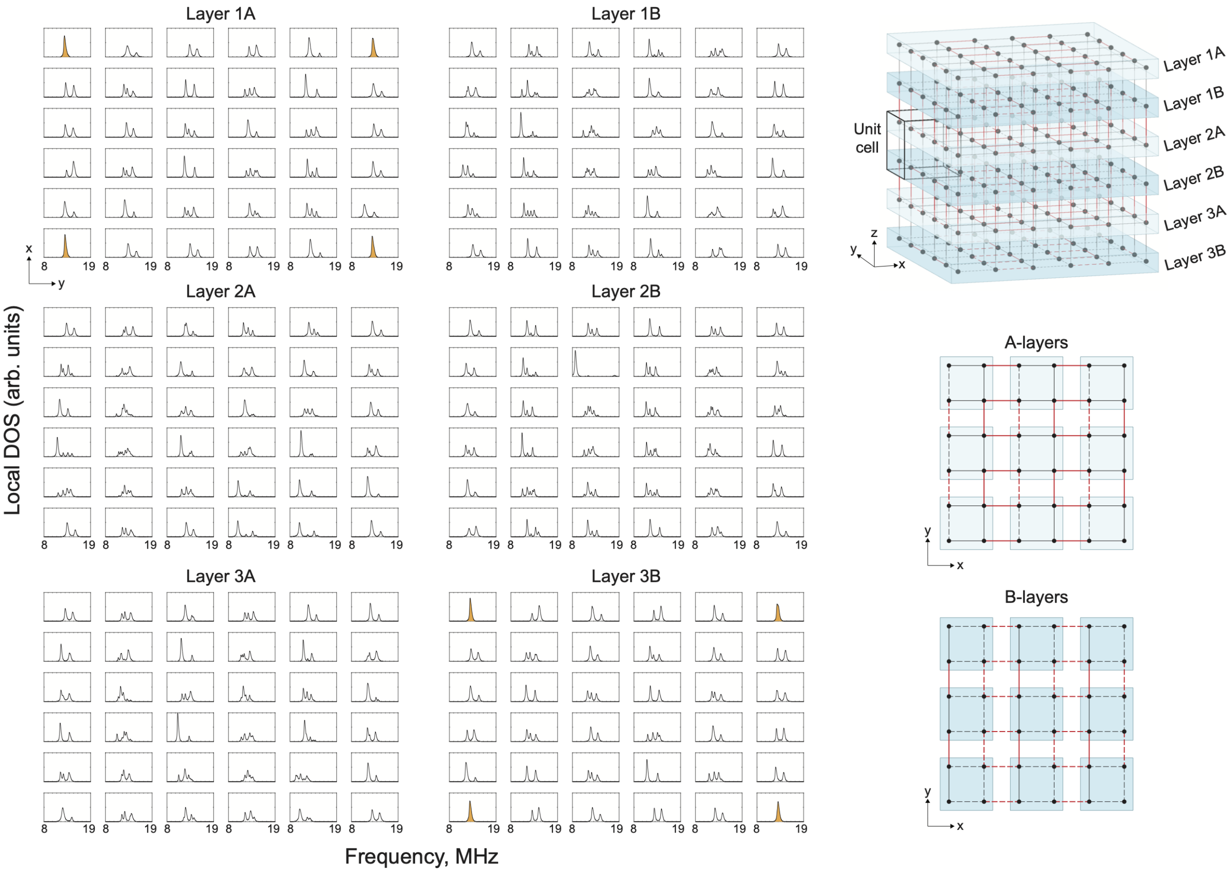

Figure S10: Measured local DOS at each site for a $3 \times 3 \times 3$ unit-cell octupole TCI with open boundaries. These measurements correspond to main manuscript Fig. 2f. The organization of the grid follows the topology on the right, the red (black) lines denote strong (weak) coupling, and the dashed lines denote negative coupling. In the DOS measurements, sites that are anticipated to host a corner mode or bound state are highlighted in yellow.

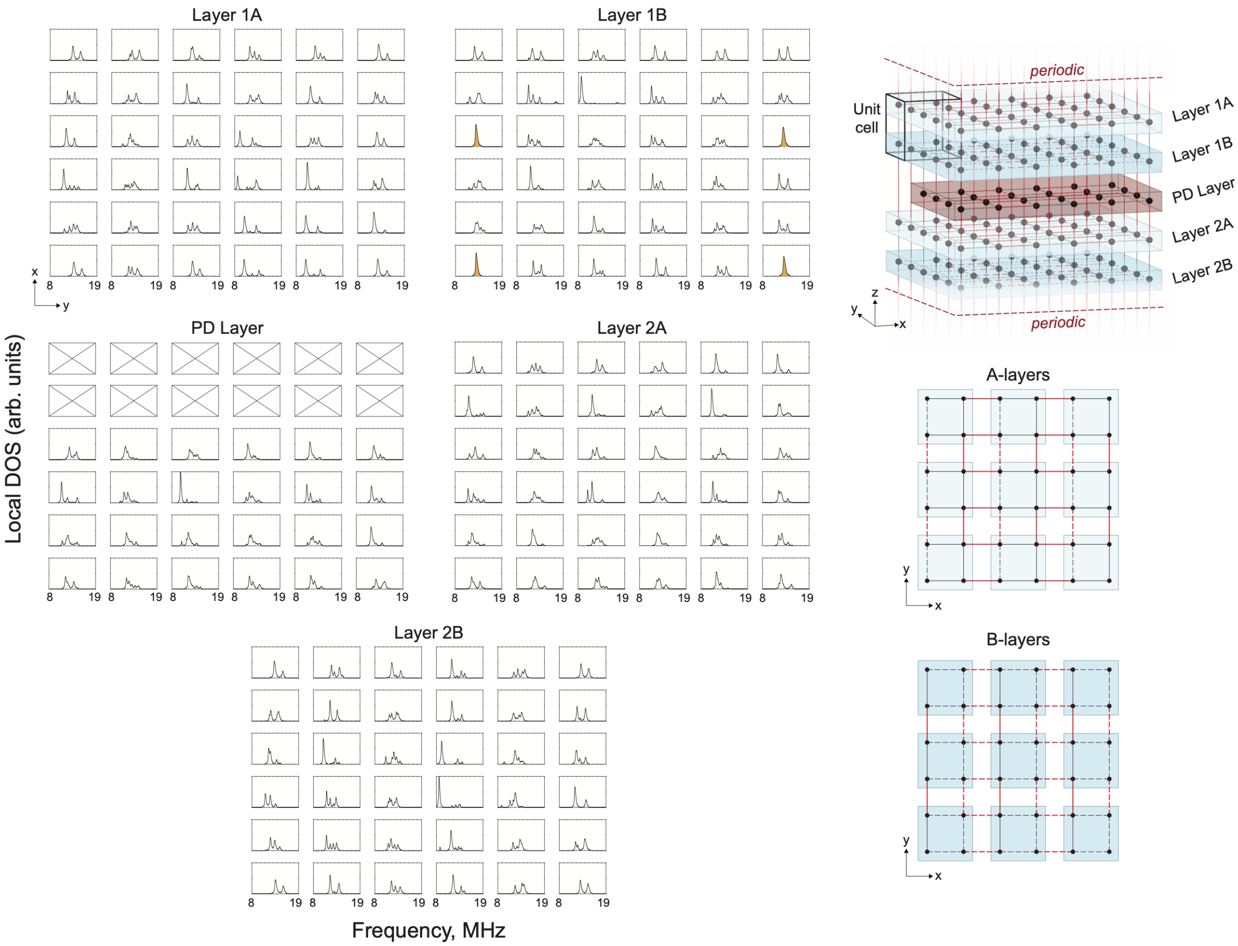

Figure S11: Measured local DOS at each site for a $3 \times 3 \times 2$ unit-cell octupole TCI with an bulk-terminated partial dislocation. These measurements correspond to main manuscript Fig. 4a. The defect is realized as 3×2 unit-cell 'A-layer' quadrupolar TCI. The organization of the grid follows the topology on the right, the red (black) lines denote strong (weak) coupling, and the dashed lines denote negative coupling. In the DOS measurements, sites that are anticipated to host a corner mode or bound state are highlighted in yellow.

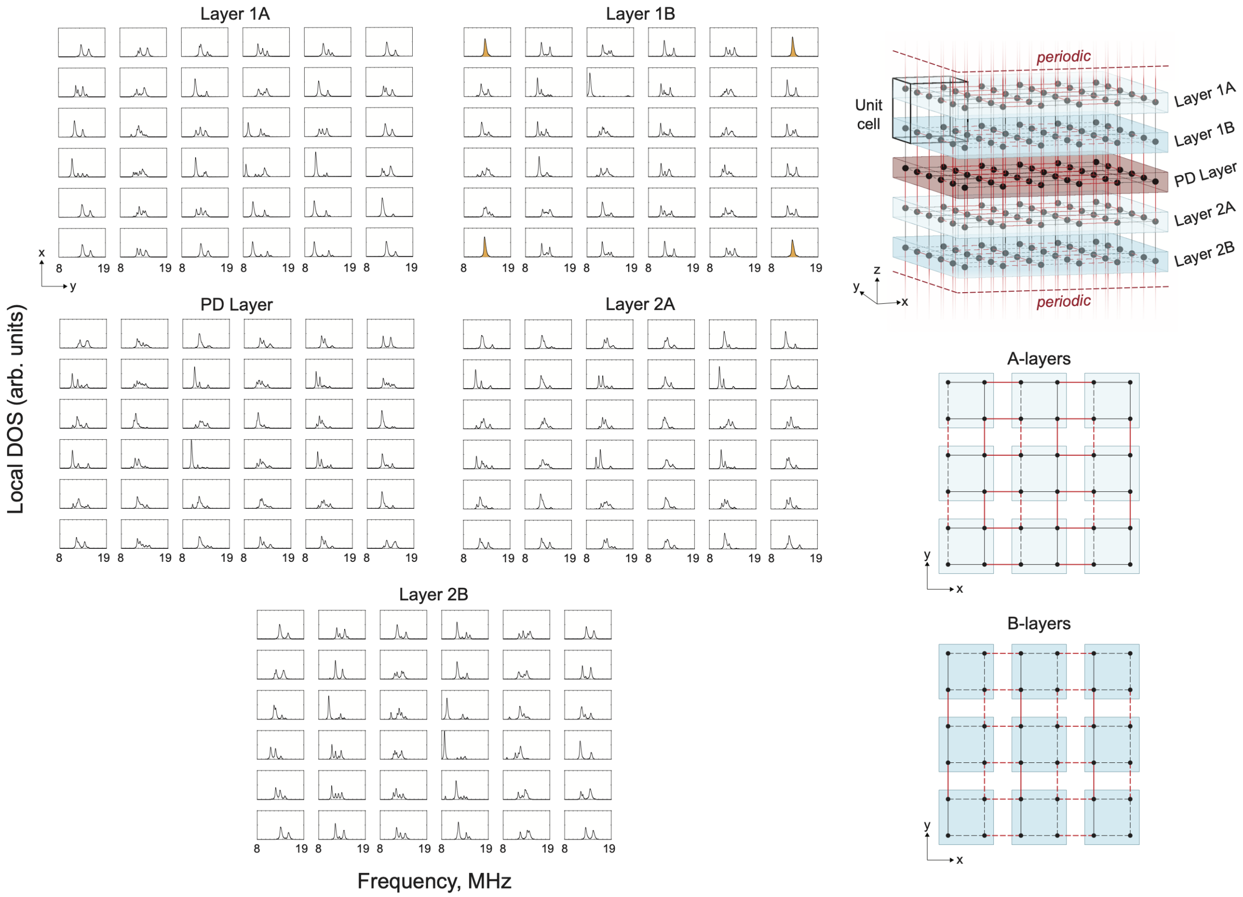

Figure S12: Measured local DOS at each site for a $3 \times 3 \times 2$ unit-cell octupole TCI with a boundary-terminated partial dislocation. The defect is realized as an 'A-layer' quadrupolar TCI plane. These measurements correspond to main manuscript Fig. 4b. The organization of the grid follows the topology on the right, the red (black) lines denote strong (weak) coupling, and the dashed lines denote negative coupling. In the DOS measurements, sites that are anticipated to host a corner mode or bound state are highlighted in yellow.

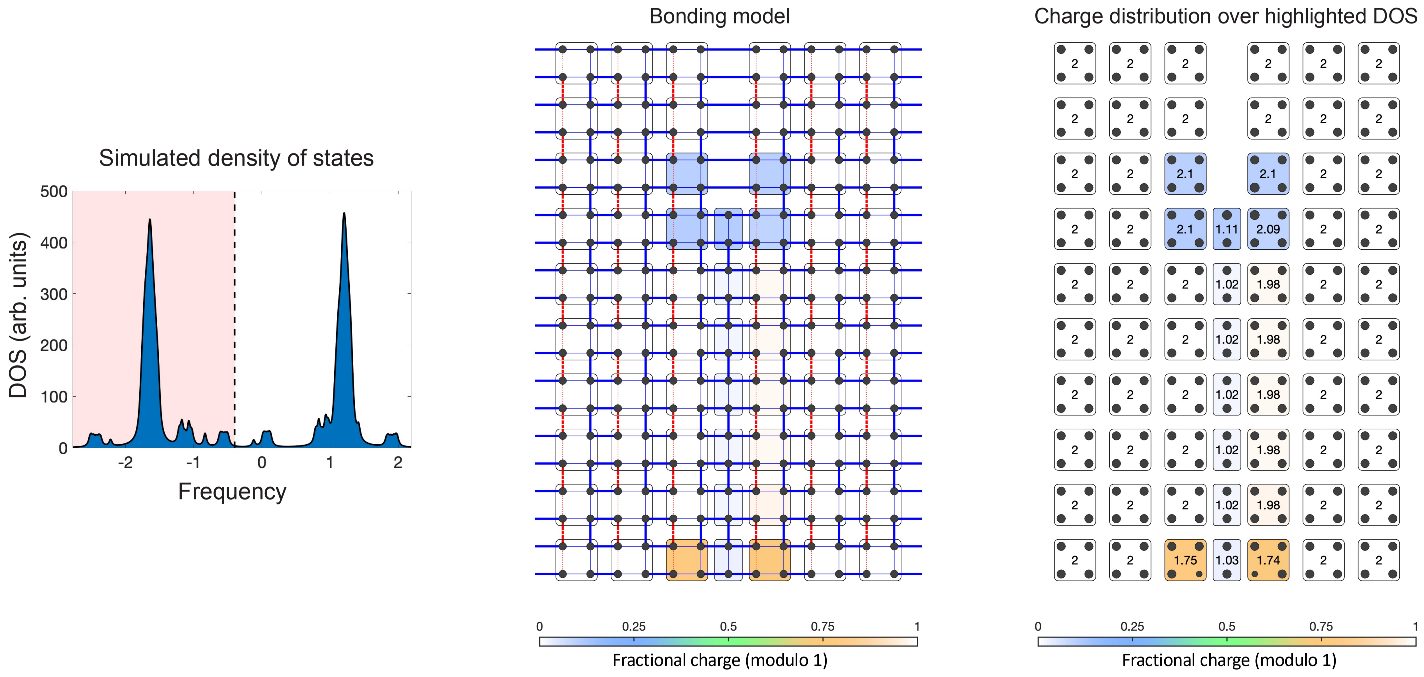

Figure S13: Simulated DOS and charge distribution within a quadrupole high-order topological insulator with partial dislocation (PD). We examine both bulk- and boundary-terminated PDs. The left and right boundaries for the system are made periodic, while the top and bottom boundaries are terminated. This simulation represents a larger lattice example of the experiment shown in Fig. 3 but with symmetric bonding near the PD terminations. The density of states is integrated over the shaded region to show the charge distribution with states filled up to a selected Fermi level. Unit cells of the structure are then color coded depending on the modulo 1 fractional charge contained within them. We can readily observe the fractional charge associated with the topology of the PD-HOTI boundary which is quantized in units of $1/2$ even though it is distributed across multiple unit cells. There is a small (but well understood) left-to-right asymmetry caused by the asymmetric position of the negative coupling in the quadrupole unit cells.

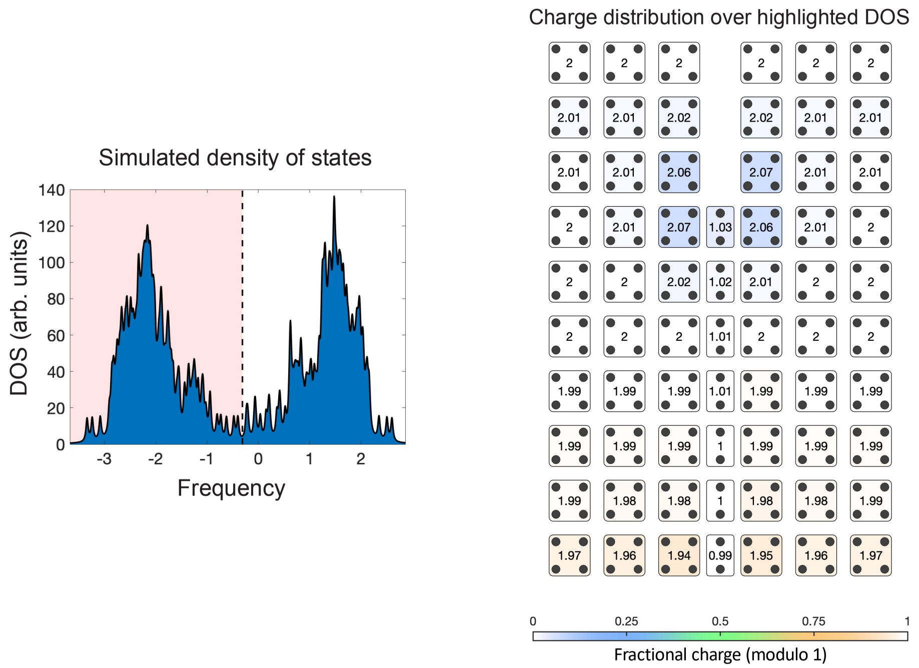

Figure S14: Similar to Fig. S13 but with increased intra-cell coupling that results in a very small band gap. We observe that the charge spreads out to more unit cells, but the total quantized fractional charge does not change in the vicinity of the PD terminations.

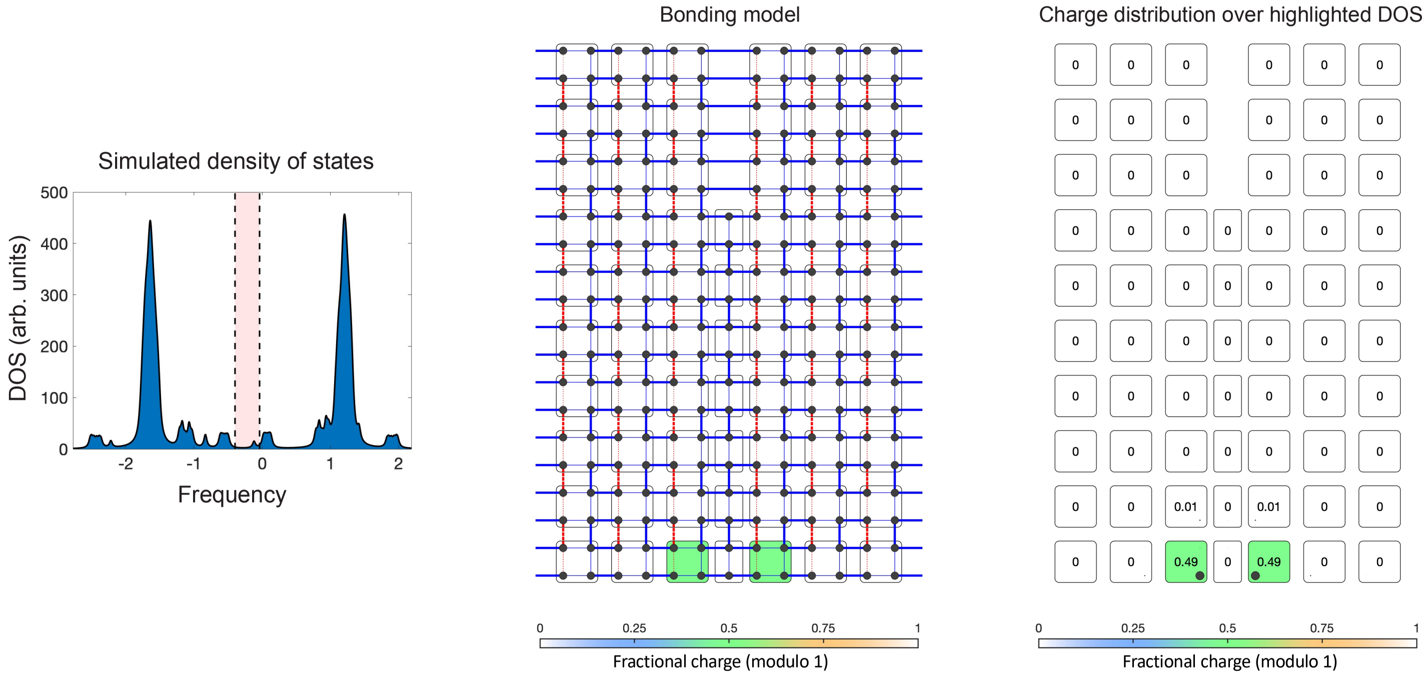

Figure S15: The case of Fig. S13 visualized within the highlighted band gap. Even though in Fig. S13 the PD reveals the high-order topology via the quantized fractional charge, here we are only able to see one localized state in the band gap. In Fig. S16 and Fig. S17 we will examine a method to bring modes at both PD terminations into the gap.

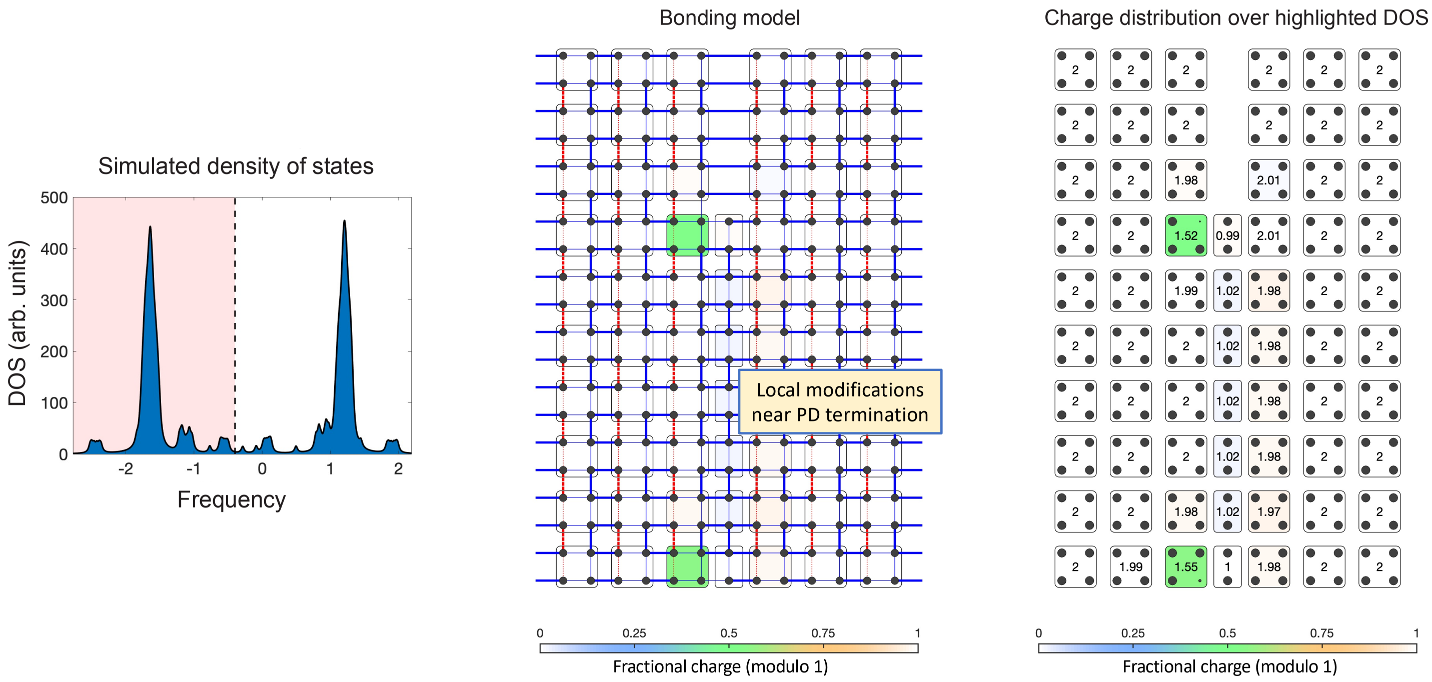

Figure S16: Starting from Fig. S13, we now modify the local bonds in the vicinity of the PD terminations (matching Fig. 3) to isolate the charge anomaly onto single sites. The DOS integrated over the lower bands now shows an even clearer signature of the $^{1}/_{2}$ quantized fractional charge.

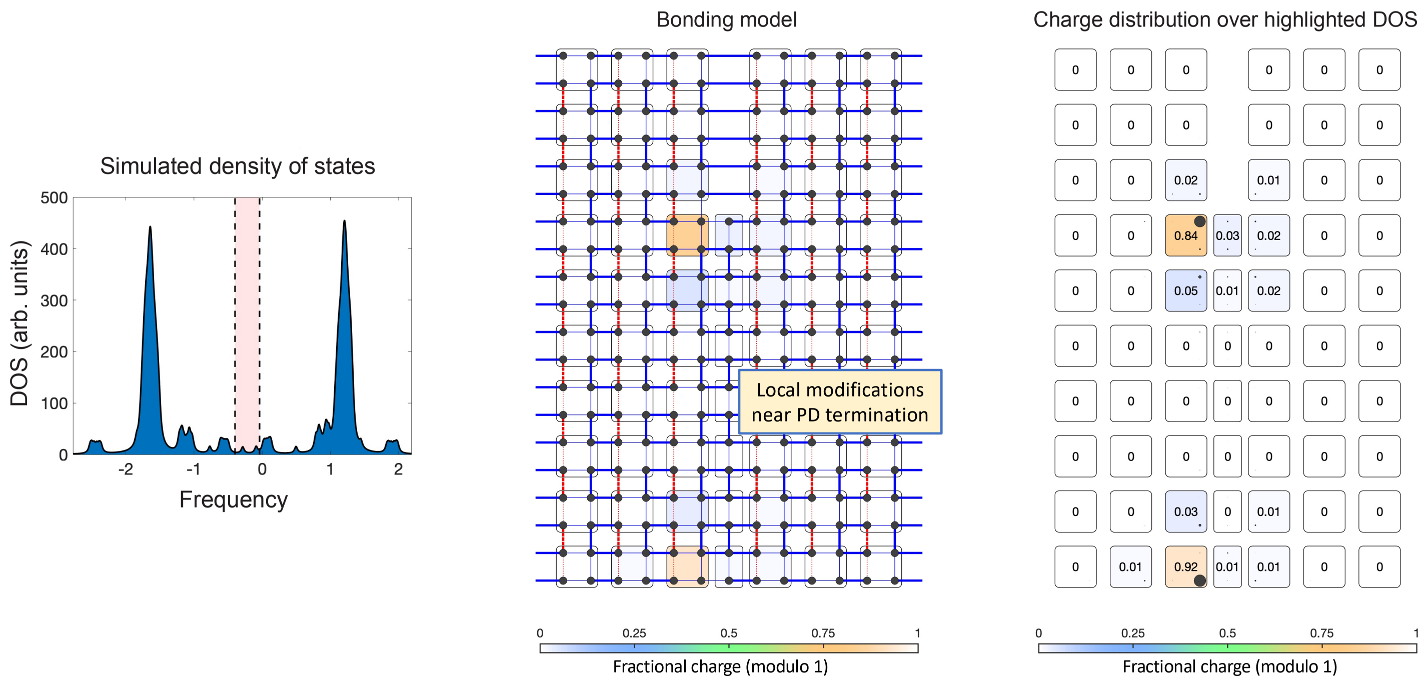

Figure S17: The case of Fig. S16 visualized within the identified band gap. We are now able to see the unit charge belonging to in-gap localized states at both terminations of the PD.
